# Poverty Status and Childhood Asthma in White and Black Families: National Survey of Children’s Health

**DOI:** 10.3390/healthcare6020062

**Published:** 2018-06-12

**Authors:** Shervin Assari, Maryam Moghani Lankarani

**Affiliations:** 1 Department of Psychology, University of California, Los Angeles (UCLA), Los Angeles, CA 90095, USA; 2 Department of Psychiatry, University of Michigan, Ann Arbor, MI 48104, USA; lankaranii@yahoo.com; 3 Center for Research on Ethnicity, Culture and Health, School of Public Health, University of Michigan, Ann Arbor, MI 90095, USA

**Keywords:** socioeconomic status, poverty, income, ethnic groups, Blacks, ethnicity, asthma

## Abstract

*Background:* Living above the poverty line reduces the risk of physical illnesses, including childhood asthma (CA). Minorities’ Diminished Return theory, however, suggests that the protective effects of socioeconomic status (SES) on health are weaker for racial minorities than White families. It is unknown whether the association between SES and CA differs for White and Black families. *Aims:* Using a national sample, the current study compared Black and White families for the association between living above the poverty line and CA. *Methods:* Data came from the National Survey of Children’s Health (NSCH), 2003–2004, a national telephone survey. A total of 86,537 Black or White families with children (17 years old or younger) were included in the study. This sample was composed of 76,403 White (88.29%) and 10,134 Black (11.71%) families. Family SES (living above the poverty line) was the independent variable. The outcome was CA, reported by the parent. Age, gender, and childhood obesity were the covariates. Race was conceptualized as the moderator. A number of multivariable logistic regressions were used in the pooled sample and specific to each race for data analysis. *Results:* In the pooled sample, living above the poverty line was associated with lower odds of CA. An interaction was found between race and living above the poverty line on odds of CA, indicating a smaller association for Black compared to White families. Although race-stratified logistic regressions showed negative associations between living above the poverty line and CA in both White and Black families, the magnitude of this negative association was larger for White than Black families. *Conclusions:* The health gain from living above the poverty line may be smaller for Black than White families. Due to the existing Minorities’ Diminished Return, policies that merely reduce the racial gap in SES may not be sufficient in eliminating racial health disparities in the United States. Public policies must go beyond reducing poverty to address structural and environmental risk factors that disproportionately impact Blacks’ health. Policies should help Black families gain health as they gain upward social mobility. As they are more likely to face societal and structural barriers, multi-level interventions are needed for the health promotion of Blacks.

## 1. Introduction

The protective effects of socioeconomic status (SES) on health [1,2,3,4,5,6,7,8] are not equal across racial groups [9,10]. While high family SES, such as family income and parental education, are protective overall [11,12], and low SES, financial strain, and poverty may partially explain why racial minorities suffer from worse childhood health [13], the smaller health gain from SES among minorities may be another mechanism by which racial disparities in health exist [9,10]. 

According to Minorities’ Diminished Return theory [9], unequal health gain from SES is a neglected mechanism behind racial health disparities [10]. Supporting this theory [9,10], considerable research has shown that SES has stronger effects on drinking patterns [14], depressive symptoms [15], suicidality [16], chronic disease [15], and mortality [17,18,19,20] for Whites than Blacks. Either due to the extra costs of upward social mobility for Blacks compared to Whites [21,22], or high levels of discrimination among Blacks [23], SES generates less health for Blacks than Whites. In some extreme examples, high SES not only does not improve health, but also becomes a risk factor for poor health of Blacks. For instance, income was positively associated with Major Depressive Disorder (MDD) among Black boys [24] and Black men [25,26]; and high education attainment is associated with a higher risk of suicide in Black women [16] and an increase in future depressive symptoms in Black men [15].

While high SES promotes health of the general population [27,28,29], this effect is not universal across racial groups [9,10,30,31,32,33]. Racial groups vary widely in their capacity to navigate the system and translate their SES resources to tangible health outcomes [23,34,35]. Although high SES reduces exposure to risks [27,28,29], these effects are unequal across various social groups [21,22,36]. That is, the very same SES indicator, such as income, generates a smaller change in purchasing power for the economically and socially disadvantaged group, compared to the privileged group [37,38,39,40]. In other words, high SES better enhances the majorities’ access to goods and services, and the health [27,28,29,41] of Whites compared to Blacks [42]. As society treats groups by their race and skin color, the same increase in SES generates smaller leverage in material resources, human capital, and psychological assets for Blacks than Whites [43,44]. One explanation for this pattern is the extra psychological and physiological costs of upward social mobility [21,22,23] for Blacks [24,41], which minimize the health gain from high SES [42,45] in this population. Blacks may also have a higher risk of using high cost effortful coping for upward social mobility [46,47]. These mechanisms collectively suggest that SES may have a smaller effect on the health of Blacks compared to Whites [34,48], as the Minorities’ Diminished Return hypothesis has suggested [9,10]. 

Childhood asthma (CA) is the leading chronic disease for children under 18 in the United States (US) [1]. Approximately seven million children suffer from asthma in the US Significant disparities in CA exist across race and socioeconomic status (SES) groups [1]. Low SES and Black families are at higher risk for CA. Prevalence of CA is 8.2%, 9.9%, and 12.2% among families with income above 200%, between 100% and 200%, and less than 100% of the federal poverty line, respectively. The risk of hospitalization, having an emergency department visit, or death from CA are all two to four times higher in Black families, compared to White families [1]. Case et al., in 2002, showed that chronic diseases such as asthma follow the social gradient in income [49].

To better understand whether Minorities’ Diminished Return theory also explains some of the racial disparities in CA, we compared Black and White families for the negative association between living above the poverty line and CA. Although research has established the effects of race [50] and SES [51,52] on CA, very few studies have ever studied multiplicative effects of race and SES on CA [53]. So, it is still unknown whether it is race and SES or race or SES that cause CA disparities [54]. To generate generalizable results on the multiplicative effects of race and SES on CA, we used data from the National Survey of Children’s Health (NSCH), a study with a nationally representative sample of children 18 years old or younger. In line with the Minorities’ Diminished Return theory [9], we hypothesized that SES (living above the poverty line) would have a larger protective effect on CA for White compared to Black families. 

## 2. Materials and Methods

### 2.1. Design and Setting

This study used a cross-sectional design. The current study used data from the NSCH (Heights Ville, MD, USA), a nationally representative study sponsored by the National Center for Health Statistics (NCHS). NSCH was a landmark survey that generated national and state-level representative prevalence estimates for a variety of children’s health indicators [55,56,57].

### 2.2. Ethics

The NSCH study protocol was approved by the CDC’s Institutional Review Board (IRB). Adolescents’ parents/legal guardians provided informed consent. Adolescents provided assent. More information on ethical aspects of the study is available [58].

### 2.3. Sampling 

Similar to other national studies, such as the National Immunization Study [55,56,57] the NSCH sampling frame was based on the State and Local Area Integrated Telephone Survey (SLAITS) [55,56,57]. To briefly describe the study sampling procedure, trained interviewers called telephone numbers at random to identify households with at least one child under the age of 18. From eligible households, one child was randomly selected for the interview. The study also included an interview with the adult in the household who knew the most about the child’s health and well-being. After excluding participants based on race/ethnicity criteria, our analytic sample consisted of 86,537 children who were 17 years old or younger (76,403 White (88.29%) and 10,134 Black (11.71%)).

### 2.4. Data Collection

The study conducted an overall number of 102,353 interviews. All the interviewers were completed between January 2003 and July 2004 and were performed either in English or Spanish. Trained interviewers asked parents/guardians a series of questions regarding their child’s physical, emotional, and behavioral health, as well as access to health care [55,56,57].

### 2.5. Variables

The current study included the following variables: child race, child demographic factors (gender and age), family socioeconomic status (SES), and child health status (overweight, and CA). 

*Race.* For confidentiality purposes, the NSCH collected child race as White only, African American/Black only, other races, and multiple races. The current study only included Blacks and Whites [55,56,57].

*Family Poverty Status (Living Above the Poverty Line).* Interviewers asked parents/guardians about household income [55,56,57]. Income to household size was based on the Department of Health and Human Services federal poverty guidelines [55,56,57]. Living above the poverty line was defined as a dichotomous variable (1 above federal poverty level or above vs. 0 less than federal poverty level) [58,59].

*Overweight.* Overweight status was a dichotomous variable calculated based on BMI which was derived from the parent’s or guardian’s reports on the height and weight of the child. Parents and guardians were asked the following two questions: “How tall is your child now?” and “How much does your child weigh now?”; BMI based on parent-reported height and weight strongly correlates with BMI based on direct measurements of height and weight [60,61]. BMI was calculated as weight (kilograms) divided by height (meters) squared. To define overweight status, the Centers for Disease Control and Prevention (CDC) gender- and age-specific growth charts were used [62]. BMI ≥ 95th percentile was considered as overweight [62,63,64]. We operationalized the variable as a two-level categorical variable (overweight vs. non-overweight).

*Childhood Asthma (CA).* A single item was used to measure the history of CA. Parents were asked, “Has a doctor or health professional ever told you that your child has Asthma? Responses included (0) No, (1) Yes, (6) Do not know, and (7) Refused. This self-reported measure of physician diagnosis has been used in the Panel Study of Income Dynamics (PSID), as well as the Behavioral Risk Factor Surveillance System (BRFSS) state-based telephone survey. Self-reported physician diagnoses are valid and reliable self-reported measures of lifetime asthma in both children and adults [65,66].

### 2.6. Data Analysis 

*Weights.* To generate nationally representative results, the NSCH sampling weights were applied. These weights are calculated based on a base sampling weight and adjustment for multiple telephone lines per household, as well as for non-response. The weights were post-stratified so that the sum of weights for each state equals the total number of children in that state as estimated for the July 2003 US census data [55,56,57].

To account for the NSCH complex survey design (due to clustering, stratification, and non-response), we used Stata 13.0 (Stata Corp., College Station, TX, USA) to analyze the data. Taylor series approximation was used for the estimation of complex design-based standard errors (SE) and variance. All percentages, means, SEs, confidence intervals (CI), and *p* values reflect the sampling weights and are thus generalizable to nationally representative estimates. 

To describe our sample, we reported frequency tables (%) and means with 95% CIs. For bivariate analysis, a Spearman correlation test was used. We ran multiple logistic regression models, first in the pooled sample and then in Whites and Blacks. In the pooled sample, the first model only included the main effects of living above the poverty line, race, and covariates. The second model also included the race × living above the poverty line interaction term. In all models, family SES (living above the poverty line) was the independent variable; CA was the dependent variable; and age, gender, and overweight status were covariates. Race was the focal moderator. Adjusted Odds Ratio (OR), 95% CI, and associated *p* values were reported. *p* values less than 0.05 were considered significant.

## 3. Results

### 3.1. Descriptives

This analysis included 86,537 Black or White children (17 years old or younger). This sample was composed of 76,403 White (88.29%) and 10,134 Black (11.71%).

Table 1 summarizes the descriptive statistics for the pooled sample, as well as White and Black children. As this table shows, Black children were from families with a lower education and lower income, and who were at a higher risk of being overweight.

### 3.2. Bivariate Correaltions

Table 2 summarizes the bivariate associations in the pooled sample. As this table shows, parent education was negatively associated with CA among children.

### 3.3. Pooled Sample Logistic Regressions

Table 3 shows the results of two logistic regressions, one without interactions and one with race by SES interactions. Model 1 showed that in the pooled sample, living above the poverty line was negatively associated with odds of CA. Model 2 showed an interaction between the effects of race and poverty status on odds of CA, suggesting that the negative association between living above the poverty line on odds of CA was smaller for Black, compared to White, families (Table 3).

### 3.4. Race Stratified Logistic Regressions

Table 4 shows the results of two logistic regressions specific to race. Model 3 and Model 4 showed a negative association between living above the poverty line (income to need ratio) and CA for White (Model 3) and Black (Model 4) children; however, the magnitude of the negative association was larger for White than Black families.

## 4. Discussion

The current study showed two findings. First, there was an association between SES and CA in the pooled sample. Second, Blacks and Whites differed in the negative association between family SES (i.e., income to need ratio) and CA. Prevalence of CA was lower for high SES Blacks and Whites; however, this association was stronger for White than Black families. 

The first finding on the protective effect of poverty status against CA was in line with the epidemiology [67] and economics [49] literature that has shown a social and economic gradient in children’s health. This literature was reviewed and explained by Case et al., in 2002 [49]. The second finding that the very same SES indicator (living above the poverty line) shows a stronger negative association with CA for White than Black families is similar to the results of studies on the association between family SES and self-rated health, obesity, and impulse control [68,69,70]. This is partly because Black families with high educational attainment have a higher risk of staying in poverty, compared to White families [71,72].

It was only recently that Minorities’ Diminished Return was found to be valid in children [68,69], as most of the supporting literature has recruited adults [15,16] or older adults [34,42]. Although the exact mechanism for a smaller health gain of SES among Blacks is still unclear, these findings support the growing evidence that differential gains start early in life and are partially responsible for racial health disparities in childhood [69]. That is, smaller health effects of family SES on the health of Black children is one reason for worse health outcomes in Black children, compared to White children [68,71]. Further, the socially privileged majority group and the socially and economically disadvantaged minority group do not equally gain from the same SES resources.

In another related study, using data following 1781 youth from birth to age 15 from the Fragile Families and Child Wellbeing Study (FFCWS) [69], Black-White differences were found in the protective effect of family structure and family SES at birth on subsequent BMI at age 15. The study revealed race by family SES and race by family structure interactions on BMI, indicating smaller effects for Blacks compared to Whites. Race by gender stratified regressions showed the most consistent patterns of associations between family SES and future BMI for male and female Whites. Family SES and structure at birth did not protect Black males or Black females against obesity 15 years later. The study was one of the first to show that the Minorities’ Diminished Return theory also holds for youth [69]. 

The results of this study should be interpreted with caution. Our results do not suggest that Blacks are unable to efficiently use their available SES resources or turn their SES resources into tangible health outcomes. This argument has been used to blame, marginalize, and stigmatize Blacks for a low chance of upward social mobility. Despite having historically been victims of slavery, racism, and discrimination [73], their socioeconomic status and poverty has been wrongly attributed to their culture [74]. Instead, it is the social structure, segregation, and structural racism that are responsible for Minorities’ Diminished Return [9,10]. Black families face disproportionately higher rates of societal and structural barriers in their lives that may hinder their ability to gain health from any SES resource that becomes available to them. The current US social system fails Blacks by charging them extra psychological and physiological costs to climb the social ladder. In a race-aware society, the process of upward social mobility is associated with more social, psychological, and physiological costs for Blacks than Whites [21,22]. The current US system is designed to maximize the gain of the privileged group even if it may cause only minimum gains for other social groups [9,10]. This offers rationale for why the US is experiencing a stubbornly high Black-White economic gap.

The easy-to-identify trait of Black race in the US has facilitated activities that systematically force Blacks into worse environments than Whites of their same SES. These forces outside the Black community in real estate, private and public facilities, and professional services can act with virtual impunity despite efforts to control them [54,75,76,77]. Sometimes they are “unconscious behaviors” of people and institutions exercising some power or professional “gate-keeping”, which exacerbates segregation and discrimination [78,79,80]. In others, they are conscious and are defended as protection against “reverse racism” [81,82,83]. 

SES may not similarly enhance the environment for Whites and Blacks, thus high SES Black families may be at a higher risk of environmental exposures to allergens, tobacco smoke, and indoor and outdoor air pollution. Other mechanisms, such as smoking, that may be more cultural than structural, may be involved. Research has shown that education has smaller protective effects against smoking [33], which may increase the risk of CA [84]. These mechanisms should be explored in future research. 

One reason why SES may fail to show strong effects for Blacks is that high SES Blacks face high levels of interpersonal and instructional aspirations. Black families who seek new opportunities are forced to fight societal barriers that increase the costs of moving up the social ladder. One example of this is the effect of discrimination on reduced health gains that commonly follow high SES [15]. We argue that in the presence of racism and discrimination, and in a race–and–color–aware society, high aspirations may not be protective but detrimental to Blacks’ health. This is in line with the recent research suggesting that high SES may be a vulnerability factor for Black families [23,24], a finding which is replicated for adults [23] and adolescents [24]. Of course, we are not suggesting that Blacks should not have high aspirations. Instead, we argue that upward social mobility should not be associated with extra costs for minority groups, and assert that all groups should benefit equally from climbing the social ladder [21,22].

The finding on the overall protective effect of family SES against odds CA is in line previous studies on the protective effects of high SES against a wide range of health outcomes [69,85,86,87,88,89]. Low SES is a root cause of illness, and CA is not an exception to this general rule [1]. Several state-of-the-art studies have shown the well-established link between SES and health [2,3,4,5,6,7,90]. 

However, this SES gain is smaller for Blacks, a pattern that is not limited to childhood [9,10]. A study showed that education better changes the drinking habits of Whites than Blacks in older adults [42]. In the Health and Retirement Study (HRS), high income was associated with low BMI for White women and Black women, but not for White men and Black men. High educational attainment was also associated with higher physical activity and sleep quality for White men, White women, and Black women, but not Black men [34]. Among adults, education [18], employment [91], neighborhood quality [92], and social contacts [93] generate a smaller gain in life expectancy for Blacks than for Whites. All these findings are in concert and support the Minorities’ Diminished Return theory of the systematically smaller health gain of SES for Blacks than Whites [16,18,34,94].

### Limitations

Our study had a few methodological limitations. As the study used a cross-sectional design, causal conclusions are not plausible. Despite the temporal ambiguity of exposure (current poverty level) and outcome (lifetime CA prevalence), it is more plausible to conceptualize poor SES as a cause and CA as an outcome. Although CA may contribute to or be followed by greater family poverty, CA is often preceded by abysmal inner-city conditions [41,95,96,97]. This study measured CA using self-reported data. Although self-reported data are valid to measure CA [65,66], the diagnosis of CA not being confirmed to meet NHLBI or other society guidelines (bronchodilator response, etc.) is a major limitation. The study is at risk of omitted confounders. We, however, controlled for the effects of obesity which is linked to SES, as well as CA medications, particularly preventive inhaled corticosteroids [98]. This study is prone to bias due to non-classical measurement error. The poverty line is endogenous, as families have some control over what their income is (through what job they choose and how many hours they work). Overall, the poverty line is not the best SES measure as it does not adjust for cost of living, and families with the same poverty status may face different levels of financial hardship depending on the part of the country. Given that many federal and state anti-poverty programs are tied to income, there may be asymmetric bunching (with many families just below, but very few just above). To address this problem, future research may use a “donut” specification, as explained by Barreca, et al., in 2011 [99]. This approach drops families within 10% or 20% of the poverty line, to reduce measurement bias [99]. In the current study, we did not have income values but levels, so we could not use “donut” specification. In addition, the study did not collect data on parents’ race. The results may differ for Black and White children with White and non-White parents. This study was limited to Blacks and Whites only. Future research should test if other minority groups such as Hispanics, Indian Americans, immigrants, sexual minorities, and other minority groups also gain less from their positive SES indicators. As shown in Table 4, the ORs for Blacks were not statistically significant. This may be partially due to the imbalanced sample sizes between Blacks and Whites. Although the NSCH data set contained variables for Hispanic ethnicity, the sample size of Hispanic Blacks was very small. So, we could not model the differences between Hispanic Whites and Hispanic Blacks. As a result, we limited our sample to non-Hispanic White and non-Hispanic Blacks. More research is also needed on the role of other ethnic groups, regions, and neighborhoods on these relationships. Future research should replicate the findings reported here among other marginalized groups, such as immigrants. Third, all the study measures were those at an individual level. There is a need for future research on contextual factors that surround Black and White families across SES levels. We also do not know if these findings hold for other SES indicators such as family structure, household size, employment, and wealth. The data were old (13 years old). The results should be replicated using other similar data sets such as PSID, NHANES, NHIS, or BRFSS. Research may also try to replicate these findings for educational attainment and other SES measures. Despite these limitations, this is one of the first studies to explore Black-White variation in the link between SES and CA. 

## 5. Conclusions

In the United States, the negative association between living above the poverty line and CA is smaller for Black families compared to their White counterparts. Future research should use longitudinal data to establish causation between SES and asthma by race. The role of structural racism, interpersonal discrimination, and societal barriers in these patterns should be explored. Public and economic policy solutions should go beyond equalizing SES and eliminate Minorities’ Diminished Return from SES, which is a neglected contributor to racial health disparities in the US Policy solutions to health disparities require jointly addressing race and SES, as race and SES do not operate independently. 

## Figures and Tables

**Table 1:** Descriptive statistics in the pooled sample and by race.

Characteristics	All (*n* = 86,537)	Whites (*n* = 76,403)	Blacks (*n* = 10,134)
	% (95% CI)	% (95% CI)	% (95% CI)
Child Race			
White	82.27 (81.69–82.83)	-	-
Black	17.73 (17.17–18.31)	-	-
Child Gender			
Male	51.08 (50.43–51.73)	51.45 (50.77–52.13)	49.38 (47.55–51.21)
Female	48.92 (48.27–49.57)	48.55 (47.87–49.23)	50.62 (48.79–52.45)
Parental Education (High school) *			
No	29.00 (28.37–29.63)	26.24 (25.61–26.89)	41.77 (39.93–43.63)
Yes	71.00 (70.37–71.63)	73.76 (73.11–74.39)	58.23 (56.37–60.07)
Family Living Outside Poverty *			
No	13.32 (12.79–13.85)	9.58 (10.08–10.08)	30.62 (28.86–32.45)
Yes	86.68 (86.15–87.21)	90.42 (89.92–90.89)	69.38 (67.55–71.14)
Child Overweight *			
No	75.84 (75.27–76.40)	78.56 (77.99–79.11)	63.23 (61.41–65.00)
Yes	24.16 (23.60–24.73)	21.44 (20.89–22.01)	36.77 (35.00–38.59)
Child Asthma *			
No	86.20 (85.74–86.65)	87.37 (86.92–87.81)	80.75 (79.20–82.22)
Yes	13.80 (13.35–14.26)	12.63 (12.19–13.08)	19.25 (17.78–20.80)
	**Mean (CI)**	**Mean (CI)**	**Mean (CI)**
Child Age (Year)	8.71 (8.65–8.77)	8.68 (8.62–8.74)	8.84 (8.67–9.00)
Income to Need Ratio *	5.38 (5.35–5.41)	5.70 (5.66–5.73)	3.90 (3.82–3.99)

* *p* < 0.05.

**Table 2:** Correlation matrix in the pooled sample (*n* = 86,537).

Characteristics	1	2	3	4	5	6	7
1 Child Race (Blacks)	1.00						
2 Child Gender (Females)	0.01 *	1.00					
3 Child Age (Year)	−0.00	−0.01 *	1.00				
4 Low Parental Education (Low SES)	−0.10 *	−0.00	−0.03	1.00			
5 Living Above the Poverty Line (High SES)	0.19 *	0.00	−0.03	−0.27 *	1.00		
6 Childhood Overweight	0.12 *	−0.07 *	−0.26 *	−0.09 *	0.09 *	1.00	
7 Childhood Asthma	0.06 *	−0.06 *	0.06	−0.02 *	0.04	0.04	1.00

* *p* < 0.05.

**Table 3:** Summary of logistic regression models in the pooled sample.

Characteristics	Model 1 (All; *n* = 86,537)			Model 2 (All; *n* = 86,537)		
	Main Effects			Main Effects + Interactions		
	OR	95% CI	*p*	OR	95% CI	*p*
Living Above the Poverty Line	0.84 **	0.74–0.95	0.008	0.73 ***	0.63–0.85	0.001
Parental Education (Low)	0.93	0.85–1.02	0.140	0.95	0.86–1.04	0.271
Child Race (Blacks)	1.55 ***	1.38–1.73	0.001	1.21	0.93–1.58	0.163
Child Gender (Females)	0.68 ***	0.63–0.73	0.001	0.68 ***	0.63–0.73	0.001
Child Age (Year)	1.04 ***	1.03–1.05	0.001	1.04 ***	1.03–1.05	0.001
Childhood Overweight	1.35 ***	1.23–1.49	0.001	1.35 ***	1.22–1.48	0.001
Low Parental Education × Race	-	-	-	0.95	0.75–1.19	0.635
Living Above the Poverty Line × Race	-	-	-	1.41 *	1.08–1.86	0.013
Intercept	0.19 ***	0.16–0.23	0.001	0.22 ***	0.17–0.27	0.001

Outcome: Childhood Asthma, Confidence Interval (CI); * *p* < 0.05, ** *p* < 0.01, *** *p* < 0.001.

**Table 4:** Summary of logistic regression models by race.

Characteristics	Model 3 (Whites; *n* = 76,403)			Model 4 (Blacks; *n* = 10,134)		
	OR	95% CI	*p*	OR	95% CI	*p*
Living Above the Poverty Line	0.73 ***	0.63–0.84	0.001	1.05	0.84–1.32	0.679
Low Parental Education	0.94	0.85–1.04	0.242	0.90	0.74–1.11	0.339
Child Gender (Females)	0.66 ***	0.61–0.72	0.001	0.73 **	0.60–0.89	0.002
Child Age (Year)	1.05 ***	1.04–1.06	0.001	1.01	0.99–1.03	0.436
Childhood Overweight	1.36 ***	1.22–1.51	0.001	1.28 *	1.03–1.58	0.026
Intercept	0.20 ***	0.16–0.26	0.001	0.32 ***	0.20–0.50	0.001

Outcome: Childhood Asthma, Confidence Interval (CI); * *p* < 0.05, ** *p* < 0.01, *** *p* < 0.001.

## References

[1] American Lung Association (ALA) Socioeconomic and Racial Asthma Disparities in Asthma. www.lung.org/local-content/illinois/documents/socioeconomic-asthma-disparities.pdf.

[2] Mirowsky J., Ross C.E. (2003). Education, Social Status, and Health.

[3] Bowen M.E., González H.M. (2010). Childhood socioeconomic position and disability in later life: Results of the health and retirement study. Am. J. Public Health.

[4] Herd P., Goesling B., House J.S. (2007). Socioeconomic position and health: The differential effects of education versus income on the onset versus progression of health problems. J. Health Soc. Behav..

[5] Kim J. (2008). Intercohort trends in the relationship between education and health: Examining physical impairment and depressive symptomatology. J. Aging Health.

[6] Van de Mheen H., Stronks K., Looman C.W.N., Mackenbach J.P. (1998). Does childhood socioeconomic status influence adult health through behavioural factors?. Int. J. Epidemiol..

[7] Leopold L., Engelhardt H. (2013). Education and physical health trajectories in old age. Evidence from the Survey of Health, Ageing and Retirement in Europe (SHARE). Int. J. Public Health.

[8] Johnson-Lawrence V.D., Griffith D.M., Watkins D.C. (2013). The effects of race, ethnicity and mood/anxiety disorders on the chronic physical health conditions of men from a national sample. Am. J. Men’s Health.

[9] Assari S. (2017). Unequal gain of equal resources across racial groups. Int. J. Health Policy Manag..

[10] Assari S. (2018). Health Disparities Due to Minorities Diminished Return: Policy Solutions. Soc. Issues Policy Rev..

[11] Alaimo K., Olson C.M., Frongillo E.A., Briefel R.R. (2001). Food insufficiency, family income, and health in US preschool and school-aged children. Am. J. Public Health.

[12] Shah C.P., Kahan M., Krauser J. (1987). The health of children of low-income families. Can. Med. Assoc. J..

[13] Chen E. (2004). Why socioeconomic status affects the health of children: A psychosocial perspective. Curr. Dir. Psychol. Sci..

[14] Hummer R.A., Lariscy J.T. (2011). Educational attainment and adult mortality. International Handbook of Adult Mortality.

[15] Assari S. (2016). Combined Racial and Gender Differences in the Long-Term Predictive Role of Education on Depressive Symptoms and Chronic Medical Conditions. J. Racial Ethn. Health Disparities.

[16] Assari S. (2015). Ethnic and Gender Differences in Additive Effects of Socio-economics, Psychiatric Disorders, and Subjective Religiosity on Suicidal Ideation among Blacks. Int. J. Prev. Med..

[17] Hayward M.D., Hummer R.A., Sasson I. (2015). Trends and group differences in the association between educational attainment and US adult mortality: Implications for understanding education’s causal influence. Soc. Sci. Med..

[18] Assari S., Lankarani M.M. (2016). Race and Urbanity Alter the Protective Effect of Education but not Income on Mortality. Front. Public Health.

[19] Backlund E., Sorlie P.D., Johnson N.J. (1999). A comparison of the relationships of education and income with mortality: The National Longitudinal Mortality Study. Soc. Sci. Med..

[20] Everett B.G., Rehkopf D.H., Rogers R.G. (2013). The Nonlinear Relationship between Education and Mortality: An Examination of Cohort, Race/Ethnic, and Gender Differences. Popul. Res. Policy Rev..

[21] Fuller-Rowell T.E., Doan S.N. (2010). The social costs of academic success across ethnic groups. Child Dev..

[22] Fuller-Rowell T.E., Curtis D.S., Doan S.N., Coe C.L. (2015). Racial disparities in the health benefits of educational attainment: A study of inflammatory trajectories among African American and white adults. Psychosom. Med..

[23] Hudson D.L., Bullard K.M., Neighbors H.W., Geronimus A.T., Yang J., Jackson J.S. (2012). Are benefits conferred with greater socioeconomic position undermined by racial discrimination among African American men?. J. Mens Health.

[24] Assari S., Caldwell C.H. (2017). High Risk of Depression in High-Income African American Boys. J. Racial Ethn. Health Disparities.

[25] Hudson D.L., Neighbors H.W., Geronimus A.T., Jackson J.S. (2012). The relationship between socioeconomic position and depression among a US nationally representative sample of African Americans. Soc. Psychiatry Psychiatr. Epidemiol..

[26] Assari S., Caldwell C.H. (2018). Social determinants of perceived discrimination among black youth: Intersection of ethnicity and gender. Children.

[27] Phelan J.C., Link B.G., Tehranifar P. (2010). Social conditions as fundamental causes of health inequalities: Theory, evidence, and policy implications. J. Health Soc. Behav..

[28] Link B.G., Phelan J. (2010). Social conditions as fundamental causes of health inequalities. Handbook Med. Sociol..

[29] Link B., Phelan J. (1995). Social conditions as fundamental causes of disease. J. Health Soc. Behav..

[30] Assari S. (2017). Social Determinants of Depression: The Intersections of Race, Gender, and Socioeconomic Status. Brain Sci..

[31] Assari S. (2018). Socioeconomic Status and Self-Rated Oral Health; Diminished Return among Hispanic Whites. Dent. J..

[32] Assari S. (2018). High Income Protects Whites but Not African Americans against Risk of Depression. Healthcare.

[33] Assari S., Mistry R. (2018). Educational Attainment and Smoking Status in a National Sample of American Adults; Evidence for the Blacks’ Diminished Return. Int. J. Environ. Res. Public Health.

[34] Assari S., Nikahd A., Malekahmadi M.R., Lankarani M.M., Zamanian H. (2016). Race by Gender Group Differences in the Protective Effects of Socioeconomic Factors against Sustained Health Problems across Five Domains. J. Racial Ethn. Health Disparities.

[35] Hudson D.L. (2009). Race, Socioeconomic Position and Depression: The Mental Health Costs of Upward Mobility. Doctoral Dissertation.

[36] Keil J.E., Sutherland S.E., Knapp R.G., Tyroler H.A. (1992). Does equal socioeconomic status in black and white men mean equal risk of mortality?. Am. J. Public Health.

[37] Cooper R.S. (1993). Health and the social status of blacks in the United States. Ann. Epidemiol..

[38] Williams D.R., Collins C. (2001). Racial residential segregation: A fundamental cause of racial disparities in health. Public Health Rep..

[39] Williams D.R., Yu Y., Jackson J.S., Anderson N.B. (1997). Racial differences in physical and mental health: Socio-economic status, stress and discrimination. J. Health Psychol..

[40] Williams D.R., Neighbors H.W., Jackson J.S. (2003). Racial/ethnic discrimination and health: Findings from community studies. Am. J. Public Health.

[41] Brunello G., Fort M., Schneeweis N., Winter-Ebmer R. (2016). The Causal Effect of Education on Health: What Is the Role of Health Behaviors?. Health Econ..

[42] Assari S., Lankarani M.M. (2016). Education and Alcohol Consumption among Older Americans; Black-White Differences. Front. Public Health.

[43] Juhn Y.J., Beebe T.J., Finnie D.M., Sloan J., Wheeler P.H., Yawn B., Williams A.R. (2011). Development and initial testing of a new socioeconomic status measure based on housing data. J. Urban Health.

[44] Ross C.E., Wu C.L. (1995). The links between education and health. Am. Social. Rev..

[45] Montez J.K., Hummer R.A., Hayward M.D. (2012). Educational attainment and adult mortality in the United States: A systematic analysis of functional form. Demography.

[46] Tyson K., Darity W., Castellino D.R. (2005). It’s not “a black thing”: Understanding the burden of acting white and other dilemmas of high achievement. Am. Sociol. Rev..

[47] Neighbors H.W., Njai R., Jackson J.S. (2007). Race, ethnicity, John Henryism, and depressive symptoms: The national survey of American life adult reinterview. Res. Hum. Dev..

[48] Montez J.K., Hummer R.A., Hayward M.D., Woo H., Rogers R.G. (2011). Trends in the educational gradient of US adult mortality from 1986 through 2006 by race, gender, and age group. Res. Aging.

[49] Case A., Darren L., Christina P. (2002). Economic Status and Health in Childhood: The Origins of the Gradient. Am. Econ. Rev..

[50] Pearlman D.N., Zierler S., Meersman S., Kim H.K., Viner-Brown S.I., Caron C. (2006). Race disparities in childhood asthma: Does where you live matter?. J. Natl. Med. Assoc..

[51] Thakur N., Oh S.S., Nguyen E.A., Martin M., Roth L.A., Galanter J., Gignoux C.R., Eng C., Davis A., Meade K. (2013). Socioeconomic status and childhood asthma in urban minority youths. The GALA II and SAGE II studies. Am. J. Respir. Crit. Care Med..

[52] Carroll K. (2013). Socioeconomic status, race/ethnicity, and asthma in youth. Am. J. Respir. Crit. Care Med..

[53] Smith L.A., Hatcher-Ross J.L., Wertheimer R., Kahn R.S. (2005). Rethinking race/ethnicity, income; and childhood asthma: Racial/ethnic disparities concentrated among the very poor. Public Health Rep..

[54] Feagin J.R. (1999). Excluding blacks and others from housing: The foundation of white racism. Cityscape.

[55] Blumberg S.J., Foster E.B., Frasier A.M., Satorius J., Skalland B.J., Nysse-Carris K.L., Morrison H.M., Chowdhury S.R., Connor K.S. (2012). Design and operation of the National Survey of Children’s Health, 2007. Vital Health Stat..

[56] Van Dyck P., Kogan M.D., Heppel D., Blumberg S.J., Cynamon M.L., Newacheck P.W. (2004). The National Survey of Children’s Health: A new data resource. Matern Child Health J..

[57] Bramlett M.D., Blumberg S.J. (2007). Family structure and children’s physical and mental health. Health Aff..

[58] National Survey of Children’s Health CATI Instrument. https://ftp.cdc.gov/pub/health_statistics/nchs/slaits/nsch07/1a_Survey_Instrument_English/NSCH_Questionnaire_052109.pdf.

[59] Wallace S.P., Padilla-Frausto D.I., Smith S.E. (2010). Older adults need twice the federal poverty level to make ends meet in California. Policy Brief UCLA Cent Health Policy Res..

[60] Spencer E.A., Appleby P.N., Davey G.K., Key T.J. (2002). Validity of self-reported height and weight in 4808 EPIC-Oxford participants. Public Health Nutr..

[61] Stewart A.L. (1982). The reliability and validity of self-reported weight and height. J. Chronic Dis..

[62] Taylor A.W., Dal Grande E., Gill T.K., Chittleborough C.R., Wilson D.H., Adams R.J. (2006). How valid are self-reported height and weight? A comparison between CATI self-report and clinic measurements using a large cohort study. Aust. N. Z. J. Public Health.

[63] Lang I.A., Kipping R.R., Jago R., Lawlor D.A. (2011). Variation in childhood and adolescent obesity prevalence defined by international and country-specific criteria in England and the United States. Eur. J. Clin. Nutr..

[64] Dumith S.C., FariasJúnior J.C. (2010). Overweight and obesity in children and adolescents: Comparison of three classification criteria based on body mass index. Rev. Panam. Salud Publ..

[65] Valerio M.A., Andreski P.M., Schoeni R.F., McGonagle K.A. (2010). Examining the association between childhood asthma and parent and grandparent asthma status: Implications for practice. Clin. Pediatr..

[66] Bhan N., Glymour M.M., Kawachi I., Subramanian S.V. (2014). Childhood adversity and asthma prevalence: Evidence from 10 US states (2009–2011). BMJ Open Respir. Res..

[67] Victorino C.C., Gauthier A.H. (2009). The social determinants of child health: Variations across health outcomes—A population-based cross-sectional analysis. BMC Pediatr..

[68] Assari S., Caldwell C.H., Mincy R. (2018). Family Socioeconomic Status at Birth and Youth Impulsivity at Age 15; Blacks’ Diminished Return. Children.

[69] Assari S., Thomas A., Caldwell C.H., Mincy R.B. (2018). Blacks’ Diminished Health Return of Family Structure and Socioeconomic Status; 15 Years of Follow-up of a National Urban Sample of Youth. J. Urban Health.

[70] Assari S., Caldwell C.H., Mincy R.B. (2018). Maternal Educational Attainment at Birth Promotes Future Self-Rated Health of White but Not Black Youth: A 15-Year Cohort of a National Sample. J. Clin. Med..

[71] Assari S. (2018). Parental Education Better Helps White than Black Families Escape Poverty: National Survey of Children’s Health. Economies.

[72] Assari S. (2018). Diminished Economic Return of Socioeconomic Status for Black Families. Soc. Sci..

[73] Adler N.E., Stewart J. (2009). Reducing obesity: Motivating action while not blaming the victim. Milbank Q..

[74] Jones R.K., Luo Y. (1999). The culture of poverty and African-American culture: An empirical assessment. Sociol. Perspect..

[75] Johnson J.H., Parnell A., Joyner A.M., Christman C.J., Marsh B. (2004). Racial apartheid in a small North Carolina town. Rev. Black Political Econ..

[76] Bass S. (2001). Policing space, policing race: Social control imperatives and police discretionary decisions. Soc. Justice.

[77] Goldberg D.T. (1998). The new segregation. Race Soc..

[78] Sewell A.A., Jefferson K.A., Lee H. (2016). Living under surveillance: Gender, psychological distress, and stop-question-and-frisk policing in New York City. Soc. Sci. Med..

[79] Pearce D.M. (1979). Gatekeepers and homeseekers: Institutional patterns in racial steering. Soc. Probl..

[80] Sewell A.A. (2015). Opening the Black Box of Segregation: Real Estate and Racial Health Disparities. Race and Real Estate.

[81] Burley D. (2005). White racial reasoning: Rational racism in the perceptions of white males. Hum. Soc..

[82] Norton M.I., Sommers S.R. (2011). Whites see racism as a zero-sum game that they are now losing. Perspect. Psychol. Sci..

[83] Chang R.S. (1995). Reverse Racism: Affirmative Action, the Family, and the Dream that is America. Hastings Const. Law Q..

[84] Farber H.J., Knowles S.B., Brown N.L., Caine L., Luna V., Qian Y., Lavori P., Wilson S.R. (2008). Secondhand tobacco smoke in children with asthma: Sources of and parental perceptions about exposure in children and parental readiness to change. Chest.

[85] Marmot M., Allen J.J. (2014). Social determinants of health equity. Am. J. Public Health.

[86] Sobal J., Stunkard A.J. (1989). Socioeconomic status and obesity: A review of the literature. Psychol. Bull..

[87] McLaren L. (2007). Socioeconomic status and obesity. Epidemiol. Rev..

[88] Ben-Shlomo Y., Kuh D. (2002). A life course approach to chronic disease epidemiology: Conceptual models, empirical challenges and interdisciplinary perspectives. Int. J. Epidemiol..

[89] Ogden C.L., Lamb M.M., Carroll M.D., Flegal K.M. Obesity and socioeconomic status in adults: United States 1988–1994 and 2005–2008. https://www.cdc.gov/nchs/data/databriefs/db50.pdf.

[90] Lynch J., Smith G.D. (2005). A life course approach to chronic disease epidemiology. Ann. Rev. Public Health.

[91] Assari S. (2017). Life Expectancy Gain Due to Employment Status Depends on Race, Gender, Education, and Their Intersections. J. Racial Ethn. Health Disparities.

[92] Assari S., Caldwell C.H. (2017). Neighborhood Safety and Major Depressive Disorder in a National Sample of Black Youth; Gender by Ethnic Differences. Children.

[93] Assari S. (2017). Whites but Not Blacks Gain Life Expectancy from Social Contacts. Behav. Sci..

[94] Assari S. (2014). The link between mental health and obesity: Role of individual and contextual factors. Int. J. Prev. Med..

[95] Andresen E.M., Malmgren J.A., Carter W.B., Patrick D.L. (1994). Screening for depression in well older adults: Evaluation of a short form of the CES-D (Center for Epidemiologic Studies Depression Scale). Am. J. Prev. Med..

[96] Antonakis J., Bendahan S., Jacquart P., Lalive R. (2010). On making causal claims: A review and recommendations. Leadersh. Q..

[97] Dawid A.P., Faigman D.L., Fienberg S.E. (2014). Fitting science into legal contexts: Assessing effects of causes or causes of effects?. Sociol. Methods Res..

[98] Scholtens S., Wijga A.H., Seidell J.C., Brunekreef B., de Jongste J.C., Gehring U., Smit H.A. (2009). Overweight and changes in weight status during childhood in relation to asthma symptoms at 8 years of age. J. Allergy Clin. Immunol..

[99] Barreca A.I., Guldi M., Lindo J.M., Waddell G.R. (2011). Saving babies? Revisiting the effect of very low birth weight classification. Q. J. Econ..

